# A novel framework to predict ADHD symptoms using irritability in adolescents and young adults with and without ADHD

**DOI:** 10.3389/fpsyt.2024.1467486

**Published:** 2025-02-12

**Authors:** Saeedeh Komijani, Dipak Ghosal, Manpreet K. Singh, Julie B. Schweitzer, Prerona Mukherjee

**Affiliations:** ^1^ Department of Computer Science, University of California, Davis, Davis, CA, United States; ^2^ Department of Psychiatry and Behavioral Sciences, University of California, Davis, Davis, CA, United States; ^3^ MIND Institute, University of California, Davis, Davis, CA, United States

**Keywords:** ADHD, irritability, adolescents, young adults, symptom prediction, hierarchical clustering, machine learning, random forest

## Abstract

**Background:**

Attention Deficit Hyperactivity Disorder (ADHD) is a common neurodevelopmental disorder in children and adolescents characterized by persistent patterns of hyperactivity, impulsivity, and inattentiveness. ADHD persists for many into adulthood. While irritability is not a diagnostic symptom of ADHD, temper outbursts and irritable moods are common in individuals with ADHD. However, research on the association between irritability and ADHD symptoms in adolescents and young adults remains limited.

**Method:**

Prior research has used linear regression models to examine longitudinal relations between ADHD and irritability symptoms. This method may be impacted by the potential presence of highly colinear variables. We utilized a hierarchical clustering technique to mitigate these collinearity issues and implemented a non-parametric machine learning (ML) model to predict the significance of symptom relations over time. Our data included adolescents (N=148, 54% ADHD) and young adults (N=124, 42% ADHD) diagnosed with ADHD and neurotypical (NT) individuals, evaluated in a longitudinal study.

**Results:**

Results from the linear regression analysis indicate a significant association between irritability at time-point 1 (T1) and hyperactive-impulsive symptoms at time-point 2 (T2) in adolescent females (β=0.26, p-value < 0.001), and inattentiveness at T1 with irritability at T2 in young adult females (β=0.49, p-value < 0.05). Using a non-parametric-based approach, employing the Random Forest (RF) method, we found that among both adolescents and young adults, irritability in adolescent females significantly contributes to predicting impulsive symptoms in subsequent years, achieving a performance rate of 86%.

**Conclusion:**

Our results corroborate and extend prior findings, allowing for an in-depth examination of longitudinal relations between irritability and ADHD symptoms, namely hyperactivity, impulsivity, and inattentiveness, and the unique association between irritability and ADHD symptoms in females.

## 1 Introduction

Attention Deficit Hyperactivity Disorder (ADHD) is a common neurodevelopmental disorder, with hyperactivity, impulsivity, and inattentiveness as key diagnostic symptoms (1–3). ADHD presentation type may vary across individuals and evolve over time (1–4).

ADHD is highly prevalent with worldwide rates of approximately 7.2% in children and 3.4% in adults. The National Survey of Children’s Health (NSCH) in the United States reports 9.8% of children and 4.4% of adults reporting a lifetime diagnosis of ADHD between 2016 and 2019. Thus, the current prevalence of ADHD in adults is estimated to be 4.4%. Among these, ADHD is more common in boys than in girls (5, 6) though the gap narrows in adulthood with women almost as likely as men to meet the diagnostic threshold (7).

### 1.1 Irritability and ADHD symptoms

Irritability is defined as an extreme negative emotional reaction to stimuli. Irritability is associated with deficits in emotional regulation and can be expressed with anger, aggression, and violence (8). Both genetic and environmental factors contribute to the etiology of irritability and its persistence or improvement over the life course (9–11). Irritability is associated with adverse social and occupational outcomes (12–14) and can predict risky behaviors such as substance use disorder, criminality, and suicidality (15–18).

Early childhood irritability predicts later functional impairments, depression and anxiety, and oppositional defiant disorder (ODD) (19, 20). Higher childhood irritability scores positively predict internalizing and externalizing problems, in later childhood and adolescence (14, 21, 22). In adolescents, irritability is associated with higher anxiety and depression, internalizing and externalizing disorders, and is later comorbid with borderline personality disorder in adulthood (23, 24).

Irritability is not a diagnostic symptom of ADHD by itself, but phasic (temper outbursts) and tonic (irritable moods) behaviors are common in individuals with ADHD (25–27). Irritability often manifests as a symptom of emotional dysregulation (ED), a condition commonly observed in both children and adults with ADHD (28–30). Mukherjee et al. (31) studied the relation between the degree of irritability and resting state functional connectivity within two groups of neurotypical (NT) and ADHD and found atypical connectivity in reward processing, cognitive control, and emotional processing regions within the ADHD group.

Despite the importance of identifying the link underlying processes between irritability and ADHD dimensions, few studies have evaluated irritability as a predictor of ADHD symptoms from childhood to adolescence (32, 33). Earlier work by our group (34) demonstrated that irritability predicted higher hyperactive/impulsive ADHD symptoms a year later in adolescents. Notably, this correlation was predominantly driven by adolescent females rather than males.

Here, we expanded upon our previous work by testing whether irritability predicts future ADHD symptoms within a larger data set of adolescents and adding a cohort of young adults to examine if the findings were valid for broader age ranges. In addition, we used a non-parametric-based machine learning technique to build a prediction model and studied the interpretability between these factors.

### 1.2 A novel framework

#### 1.2.1 Hierarchical clustering

We explored new methods to analyze our data due to the nature of the assessment measures used in diagnosing ADHD symptoms. The assessment tools used to diagnose ADHD include items on rating scales that may highly overlap in meaning and function. For example, self-regulation can refer to both the regulation of attention (inattention) and the regulation of decision-making (impulsivity). An inattentive symptom like “difficulty sustaining attention” may lead to impulsive behaviors such as “blurting out answers” (35). Inattention can also be a driving factor in hyperactivity-impulsivity symptoms in adolescents (36). Collinearity can be a concern when analyzing the data from these assessment tools, as highly correlated items may not provide sufficient unique information and can lead to inaccurate parameter estimations and misinterpretations (37). This can pose even more challenges when dealing with small sample sizes where the instances may not allow the inclusion of all predictor variables in the analysis. Hierarchical clustering is an effective approach when dealing with collinear variables within these types of datasets (38, 39). Hierarchical clustering is one of the feature engineering techniques that groups similar data points into clusters, forming a tree-like structure. This approach can be particularly helpful in psychiatric research where there are problems with a high number of variables and a low number of instances. Through clustering and visualization of the data points, meaningful insights can be obtained about relations between variables and further forming hypotheses (40).

#### 1.2.2 Machine learning (ML)

ML as a tool can assist researchers in identifying patterns and correlations between symptoms and further predict functional outcomes or longitudinal changes regarding the significance of individual symptoms. Various studies on ADHD have utilized ML models employing linear regression, linear support vector machine (SVM), and decision tree algorithms (41–43). Linear regression models and SVMs are parametric methods and make key assumptions about the data distribution. Although these models perform well with a slight violation against these assumptions, building an ML model that does not assume a specific data distribution may enhance the assessment of relations within non-linear and non-normally distributed data. Moreover, other than prediction, the explanation of causal relationships is an important aspect of psychiatric research where decision tree algorithms provide a stronger framework (44). Random forest (RF), an extension of decision tree algorithm approaches, is suitable for problems where predictor variables are large in count, highly collinear, and similar in their measurement scale. RF approaches have been used as a validation and prediction technique for both cross-sectional and longitudinal data analysis and provide a framework for the explanation of the model using features’ importance for the effect of variables on the fit of the model (45). In previous ADHD studies, RF approaches have shown promising results for the classification of ADHD-NT participants using clinical measures, identification of relations between ADHD symptoms and other disorders, and distinction of disorders that have similar symptoms to ADHD (46–48). In this study, we apply this comprehensive approach, combining clustering and ML to reveal relations and identify patterns within our dataset.

## 2 Methods

### 2.1 Participants

We used data collected from an ongoing project, Mapping Impulsivity’s Neurodevelopmental Trajectory (MINT - R01MH091068) [see references for details on diagnostic procedures (31, 34, 49–51)], which employs multi-modal neuroimaging and clinical measures to study the neurodevelopmental trajectories of impulsivity in adolescents and young adults. All participants were diagnosed by licensed psychologists with extensive experience diagnosing ADHD (JFD or JBS), using the Diagnostic and Statistical Manual of Mental Disorders 5th Edition (1). All participants were evaluated and diagnosed for ADHD or as NT using ADHD rating scales with parents completing the Conners’ Parent Rating Scale – 3 on their children (51) with supplementary information from the Teacher (Conners-3 Teacher Rating Scale—CTRS-3) for adolescent participants. Young adults rated their ADHD symptoms using the Conners’ Adult ADHD Rating Scale (CAARS) – Self version and an Observer (e.g., parent/spouse/friend). Parents (or if a parent was not available, another older relative) were asked to rate the young adults’ childhood behavior using the Barkley Adult ADHD Rating Scale–IV (Other Report) (52) to further establish the presence or absence of ADHD childhood symptoms. Diagnosis (or absence) was further established with adolescent participants and their parents completing full clinical interviews with the Diagnostic Interview Schedule for Child and Adolescents (DISC) (53) for earlier participants in the study and then the MINI International Neuropsychiatric Interview (MINI)—Kid (54) for later participants. Clinical interviews for the young adult participants used the DISC-Young Adult version or a MINI interview (55) along with the Diagnostic Interview for ADHD in Adults (DIVA) (56) so that each young adult was given a clinical interview based on the DSM, including items on the ADHD scale as the earlier version of the MINI for adults did not include an ADHD scale.

Participants were between the ages of 12 and 30 years. Participants, both ADHD and neurotypical (NT), were required to have an intellectual functioning score of 80 or higher. This was assessed using the Wechsler Intelligence Scale for Children (WISC) and the Wechsler Adult Intelligence Scale (WAIS). For participants recruited during COVID-19 pandemic restrictions (n=16), intellectual functioning was evaluated by our Ph.D. level psychologists based on educational performance reported by parents or by adult participants themselves. Criteria included no reported need for special educational services related to intellectual challenges. Participants were excluded if they had an academic learning disability, indicated by scores below 80 in reading or math assessments on the Wechsler Individual Achievement Test (WIAT). During pandemic restrictions, the assessment of academic learning disabilities was based on parent or self-report regarding the need for special educational services at school. For ADHD diagnostic criteria, the DSM-5 criteria were used to categorize participants as either NT or having ADHD Combined Presentation. A category of “subthreshold ADHD” was defined for individuals displaying fewer than nine, but more than three, symptoms of ADHD.

The study excluded participants with IQ < 80, with a lifetime history of autism spectrum disorders, any other severe mental diagnosis (depression, post-traumatic stress disorder, psychosis), specific chronic medical illness, academic learning disorders, specific or focal neurological disorder, history of substance dependence or abuse disorder currently or within past five years, contraindications for neuroimaging. Participants with ODD, a disorder that may be present in early childhood, were permitted to be in the study, however, only five participants in the study were diagnosed with ODD. The study excluded individuals with diagnosable depressive disorders as a main aim of the broader study was to follow the trajectory of the emergence of depression in later adolescence and young adulthood in relation to heightened impulsivity manifested by individuals with ADHD. Participants using non-ADHD psychotropic medications within the past year were excluded. Participants prescribed stimulant medications or atomoxetine for ADHD were permitted to enroll in the study. A licensed psychologist (JFD or JBS) reviewed all the diagnostic information to determine the final ADHD (or NT) diagnosis based on all the diagnostic information.

Informed written parental consent and child assent were obtained from all participants or consent from the adult participants themselves by trained research staff during their first encounter, before completing the psychological evaluation. Participants were compensated for their time in completing measures and rating scales at each wave. A university Institutional Review Board approved the study.

The study included a total of 272 participants, comprising 142 males and 130 females, aged 12 to 30 years. **Table 1** provides a summary of the participants’ demographics, categorized into two subgroups: adolescents (ages 12–17) and young adults (ages 18–30).

**Table 1:** Demographic Information: characteristics of adolescent and young adult groups.

	Total (%)	Adolescents (%)	Young Adults (%)
Diagnosis			
ADHD	48.61	29.67	19.04
NT	51.29	24.92	26.37
Ethnicity			
Non-Hispanic	74.45	42.70	31.75
Hispanic	23.36	10.22	13.14
Unknown	2.19	1.82	0.36
Race			
White	65.69	39.05	26.64
Black/African American	3.28	2.19	1.09
Asian	7.30	1.09	6.20
Multi race	17.88	11.31	6.57
Others or unknown	5.84	1.09	4.74
Income (Parental for youth)			
100k and above	71.14	45.77	25.37
50k-100k	14.93	7.96	6.97
Less than 50k	13.93	6.97	6.97
Parent Education			
Bachelor’s degree or higher	27.11	3.3	23.81
Some college or associate degree	27.47	10.99	16.48
High school or less	45.42	40.29	5.13

### 2.2 Measures

ADHD rating scale items were derived from the highest scores obtained from Conners’ Parent and Self-Report Scales, as well as from CAARS Self-Report and Observer (e.g., parent, spouse, friend, sibling) Rating Scales, across two distinct age groups: adolescents (ages 12–17) and young adults (ages 18–30). These data were collected as part of a longitudinal study in which clinical measures were assessed at multiple time points.

In this study, Timepoint 2 (T2) occurred approximately one to six years after Timepoint 1 (T1), with follow-up intervals averaging 1.41 years (SD = 0.24) for adolescents and 3.85 years (SD = 2.04) for young adults. At T1, the adolescent group included 94 males and 54 females, which decreased to 51 males and 33 females at T2. In the young adult group, there were 48 males and 76 females at T1, which dropped to 15 males and 31 females at T2.

We used the DSM-oriented ADHD constructs, including hyperactivity/impulsivity and inattentiveness, as derived from Conners’ rating scales for adolescents and young adults (57). Irritability items were from the Conners’ and CAARS selected based on their similarity in content with the commonly used irritability scale, the Affective Reactivity Index (ARI) (58) and included ‘1. loses temper, 2. is angry and resentful, 3. is easily annoyed, 4. has temper outbursts, 5. becomes irritable when anxious’ for adolescents and ‘1. is easily frustrated, 2. has a short fuse/hot temper, 3. is irritable, 4. throws tantrums’ for young adults.

The raw score for each symptom was calculated by summing all items within the respective subscale, with individual item scores ranging from 0 (*not true at all*) to 3 (*very true*). The total scores for the hyperactive/impulsive and inattentive scales could reach a maximum of 27. For irritability, the total score on the Conners’ scale could be as high as 15, while the CAARS irritability score had a maximum of 12 due to one fewer item compared to the Conners’ scale used for adolescents. **Table 2** provides the descriptive statistics for all participants.

**Table 2:** Descriptive statistics.

Measures Mean, (SD)		Time 1					Time 2				
		N	Age	HYIM	IA	IR	N	Age	HYIM	IA	IR
Conners’	Male	94	14.4 (1.6)	15.4 (8.2)	18.2 (8)	5.5 (3.6)	51	16.4 (1.3)	10.2 (6.7)	14.6 (7.6)	4.8 (3.4)
	Female	54	14.5 (1.6)	11.5 (7.4)	16.1 (8.6)	5.6 (4.1)	33	16.2 (1.2)	7.5 (5.4)	12.4 (7.2)	4.6 (3.8)
CAARS	Male	48	23.1 (3.3)	10.1 (6.6)	12.1 (7.6)	3.2 (2.2)	15	25.2 (2.8)	10.4 (7.2)	10.7 (7.3)	3.8 (2.4)
	Female	76	22.4 (3.0)	9.1 (6.2)	10.5 (7.5)	3.7 (2.5)	31	25.5 (2.5)	7.7 (6.0)	9.0 (7.0)	3.2 (2.6)

Age, Years; HYIM, Hyperactive-impulsive (min:0 - max:27); IA, Inattentive (min:0 – max:27); IR, Irritable [min:0 – max: 15(Conners), 12(CAARS)].

In both adolescents and young adults, the non-returners were predominantly male; however, the sex ratio remained consistent across both time points. Among adolescents, non-returners exhibited significantly higher inattentive scores at T1. While hyperactivity/impulsivity and irritability scores decreased in adolescents over time, changes in hyperactive/impulsive, inattentive, and irritable symptoms were not significant among young adults.

At T1, no significant differences were observed between sexes for irritability and inattentiveness (p-value > 0.05). However, males displayed significantly higher hyperactivity/impulsivity scores compared to females. By T2, there were no significant differences between sexes across any of the measured constructs.

We utilized an adaptation of the Peterson Puberty Development Scale (59) to assess pubertal stage. Our analysis revealed no significant differences in irritability measures between individuals in the pubertal stage and those who were not.

### 2.3 Analytic approach

Our analysis builds upon a previously published study by our group (34), which demonstrated that irritability predicts hyperactive/impulsive symptoms in adolescent females. Here, we expand upon this analysis by utilizing a larger sample size and further investigating the data at the behavioral level through hierarchical clustering and an ML approach.

To begin, we conducted a multivariate regression analysis of the irritability subscale and ADHD constructs consistent with extant methods, encompassing hyperactivity, impulsivity, and inattentiveness symptom ratings, among both adolescents and young adults. Following this analysis, we utilized ML to explore the behavior-level relations, particularly focusing on groups where irritability serves as a predictor variable of ADHD symptoms. We addressed the issue of collinearity between variables and constructed an ML prediction model based on the results of the multivariate regression analysis.

#### 2.3.1 Multi-variate regression analysis

Multi-variate regression analysis is a statistical approach that extends the concept of simple linear regression and examines the relation between two or more independent variables and a dependent variable. This technique estimates the coefficients for the independent variables in such a way that the model best fits the observed data.

In the initial step of our analysis, we performed an extensive multivariate regression assessment to investigate the relation between ADHD constructs and irritability. This analysis started with a thorough evaluation of the prerequisites for applying a multivariate regression model to our dataset. These prerequisites included the examination of linearity, homoscedasticity, and the normality of data distribution. Initially, we constructed a multivariate regression model using inattentive, hyperactive/impulsive subscale scores, and the irritability subscale across the entire participant group (N=272) spanning from T1 to T2. We assessed the residual plots for each symptom to ensure the normality of data distribution. We examined different scaling mechanisms and chose min-max scaling to make the distribution closer to normal.

Moreover, we separated the analysis into two groups, adolescents, and young adults. In each group age at T1 was considered as a covariate and potential sex-based differences were examined. Given that the follow-up interval between T1 and T2 ranged from approximately one to six years, we evaluated whether to include time elapsed as a covariate in our analyses as well. The follow-up occurred approximately 1.41 years (SD = 0.24) after T1 for adolescents and 3.85 years (SD = 2.04) for young adults. When age at T1 and the time interval between T1 and T2 were incorporated as covariates, the associations were not significant and reduced the overall model fit. As a result, these factors were excluded from the final analyses.

For our analytical framework, we employed the Sequential Equation Modeling (SEM) methodology executed using R’s Lavaan Package (60). Lavaan provides the advantage of using Full Information Maximum Likelihood (FIML) estimation to handle missing data (61). FIML allows the contribution of all available information to enhance parameters’ estimation while minimizing the potential for bias.

To improve the precision of our model’s parameter estimation and approximate a distribution that aligns more closely with normal distribution, we applied bootstrapping with 500 draws. This technique enhances the robustness of our findings and the overall validity of our analytical outcomes.

#### 2.3.2 Machine learning approach

**Figure 1:**
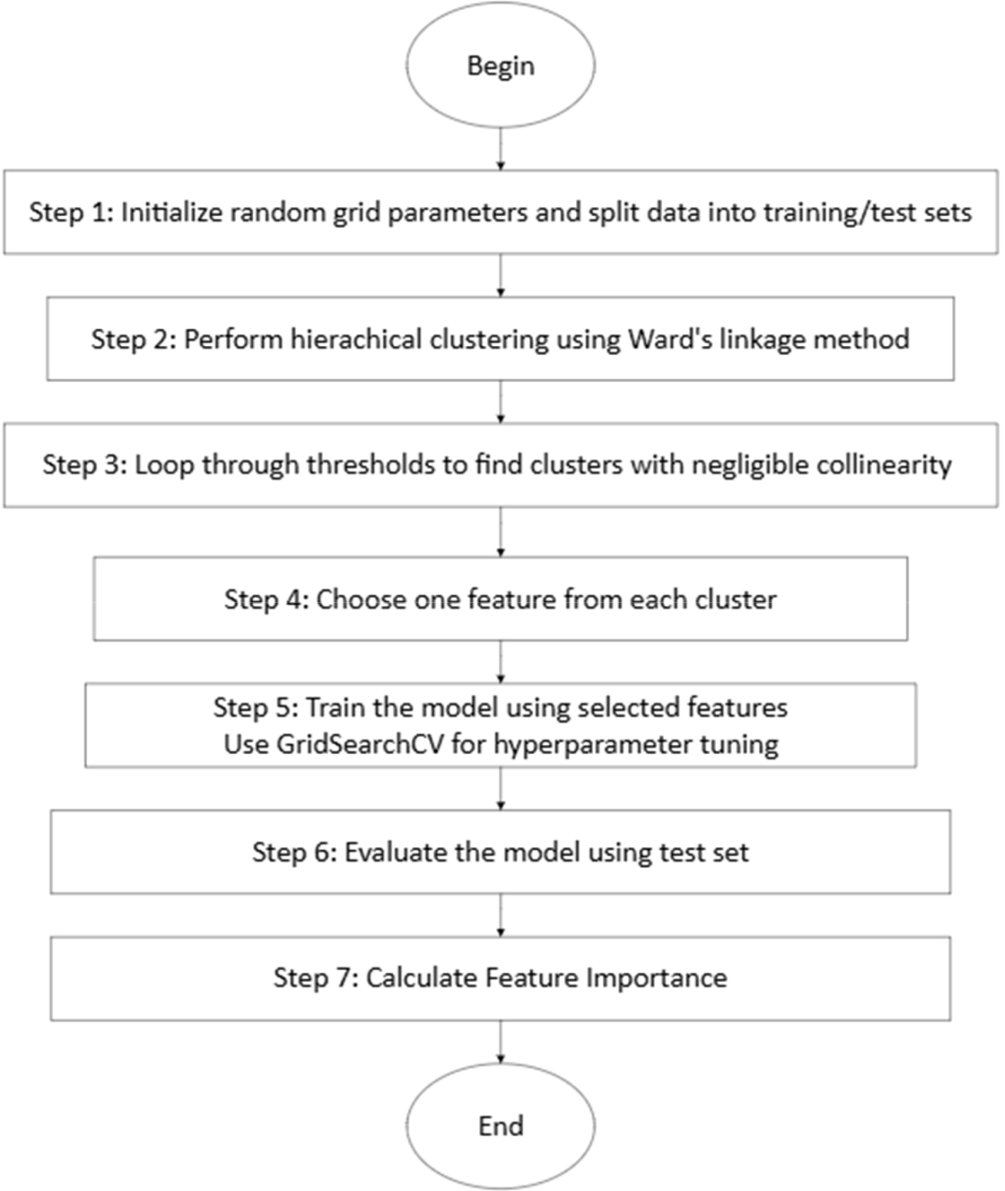
Feature clustering and model selection algorithm.

Based on the results from multivariate regression analysis, we performed a behavior-level analysis using an ML classifier for the groups where irritability was a predictor variable of ADHD symptoms in later years. **Figure 1** summarizes the algorithm for preparing the data and building the prediction model. We performed hierarchical clustering and from each cluster, we selected one representative variable. These variables together were inputted into our ML model. The input features to our model included 9 items of hyperactivity/impulsivity, 9 items of inattentiveness, and 5 (adolescents) or 4 (young adults) items of irritability at T1. These features were used as predictor variables for ADHD symptoms that revealed a significant change at T2 (based on results of multivariate regression analysis). For both inattentive and hyperactive/impulsive symptoms t-scores above 60 were labeled as 1, significant, and t-scores below 60 were labeled as 0, not significant.

To enhance the interpretability of our model and validate the results of the regression analysis, we applied our sample data to an RF classifier. RF is a supervised machine learning algorithm that is based on an ensemble learning technique called bagging (62). RF models are built using many decision trees trained by a bootstrapped dataset randomly sampled from the original data. Decision trees by themselves are easy to implement and robust to interpret and random forests have the advantage of aggregating the results of various trees to output the prediction value and gain a significant improvement in accuracy performance (63). RF is a non-parametric model that does not rely on assumptions about data distribution. It offers the advantage of identifying non-linear relations between features, setting it apart from linear regression and other machine learning models such as the Support Vector Machine (SVM). Further, using cross-validation techniques, RF can mitigate the overfitting problem for small samples that have many variables. We used Scikit-learn library in Python to implement our ML model (64).

#### 2.3.3 Multicollinearity and hierarchical clustering

To address the issue of multicollinearity among features, we used a hierarchical clustering approach based on Ward’s linkage method. Ward’s linkage method determines the distance between two clusters and is computed based on minimizing the increase in Error Sum of Squares (ESS) after merging two clusters into a single cluster. The ESS for a set of observations of a single variable X is calculated as:


$$ESS\left(X\right)=\;\sum_{i=1}^{N_{x}}{\left|x_{i}-\frac{1}{{\text{N}}_{x}}\sum_{j=1}^{N_{x}}x_{j}\;a^{n-k}\right|}^{2}$$

Where |.| represents the absolute value, $x_{i}$ and $x_{j}$ are observations of variable X, ${\text{N}}_{x}$ is the number of observations for variable X, and $a^{n-k}$ is a constant. The distance between two clusters (X and Y) is defined as:


D(X,Y)=ESS(XY)−[ESS(X)+ESS(Y)]

where XY is the combined cluster merged from two clusters X and Y, and ESS is the Error Sum of Squares as described above.

In hierarchical clustering, we define a “linkage threshold” as a criterion to determine the level of granularity. Each threshold decides at what level of distance (similarity) to stop merging clusters. Clusters that are closer than the threshold are combined, while clusters that are farther apart are not merged. The choice of threshold determines the number of clusters, the higher the threshold the lower the number of clusters. Clusters are characterized by non-significant correlations between them. We initially set the “linkage threshold” to 0, effectively considering each feature as a distinct cluster. Subsequently, we increased this threshold incrementally till there was no strong correlation between the features. We selected one feature from each of the final clusters thus obtained and used as inputs to the RF classifier.

#### 2.3.4 Model optimization and evaluation

To enhance the model’s performance, we employed GridSearchCV within scikit-learn, creating a grid of hyperparameters to identify the best parameters for the model. These hyperparameters included:

- Number of estimators: represents a set of trees trained on a subset of samples and input features.
- Maximum feature: rules the selection of features for decision-making at each node in a tree.
- Maximum depth: controls the complexity of individual trees by specifying the maximum depth of each tree.
- Minimum number of leaves: sets the minimum number of samples required in a leaf, influencing the granularity of the tree.
- Minimum number of samples’ split: regulates the partitioning of data within each leaf node.

To prevent overfitting, we applied Stratified K-Fold Cross-Validation (65) with 5 folds, ensuring that each fold maintained the same proportion of classes as the original dataset. For model evaluation, we calculated the mean and standard deviation of precision, recall, and F1 scores across iterations of the cross-validation folds and the testing dataset. Additionally, to explain the model, we used feature importance estimates, which indicate the contribution of each feature to the model’s performance.

## 3 Results

### 3.1 Multivariate linear regression

The initial multivariate regression model, designated as the baseline model, included pathways linking constructs at T1 to their corresponding constructs at T2. In this model, hyperactivity/impulsivity, inattention, and irritability at T1 served as independent variables, while the same constructs at T2 were treated as dependent variables. The baseline model was refined iteratively by adding or removing paths to optimize model fit, assessed using the Akaike Information Criterion (AIC), Bayesian Information Criterion (BIC), Root Mean Square Error of Approximation (RMSEA), Comparative Fit Index (CFI), and Tucker-Lewis Index (TLI).

In the baseline model for adolescents, significant paths were observed from each construct at T1 to its corresponding construct at T2, resulting in AIC = -423.583, BIC = -360.642, RMSEA = 0.134, CFI = 0.966, TLI = 0.915.

Subsequently, paths from “irritable” at T1 to “hyperactive-impulsive” and “inattentive” at T2 were introduced (AIC = -429.385, BIC = -360.449, RMSEA = 0.117, CFI = 0.983, and TLI = 0.935). Since the path from “irritable” to “inattentive” was not significant, it was eliminated. This resulted in a slight decrease in model fit (AIC = -429.032, BIC = -363.094, RMSEA = 0.113, CFI = 0.980, and TLI = 0.939) but made the model simpler and therefore it was removed.

Further adjustments were made by introducing paths from “hyperactive-impulsive” and “inattentive” at T1 to “irritable” at T2. However, these paths were removed as they were not significant and decreased the model fit (AIC = -427.024, BIC = -355.151, RMSEA = 0.146, CFI = 0.980, and TLI=0.899).

For young adults, we followed the same procedure. For the baseline model, we introduced paths from each construct at T1 to the corresponding construct at T2 resulting in AIC = -338.180, BIC = -278.954, RMSEA = 0.135, CFI = 0.963, and TLI = 0.907.

Then we introduced the paths from “irritable” at T1 to “hyperactive-impulsive” and “inattentive” at T2 which resulted in a decrease in model fit (AIC = -335.573, BIC = -270.706, RMSEA = 0.169, CFI = 0.961, and TLI = 0.854) thus the paths were removed.

Next, we introduced paths from “hyperactive/impulsive” and “inattentive” at T1 to “irritable” at T2. (AIC = -343.321, BIC = -278.455, RMSEA = 0.114, CFI = 0.982, and TLI=0.934). The path from “hyperactive/impulsive” to “irritable” was not significant and removing it resulted in an improvement in model fit (AIC = -345.007, BIC = -282.960, RMSEA = 0.096, CFI = 0.984, and TLI = 0.953). In this final model the path from irritable to irritable was not significant and removing it made a slight change in model fit, therefore we eliminated the path to make the model simpler (AIC = -343.244, BIC = -284.018, RMSEA = 0.107, CFI = 0.977, and TLI = 0.942).

**Figure 2:**
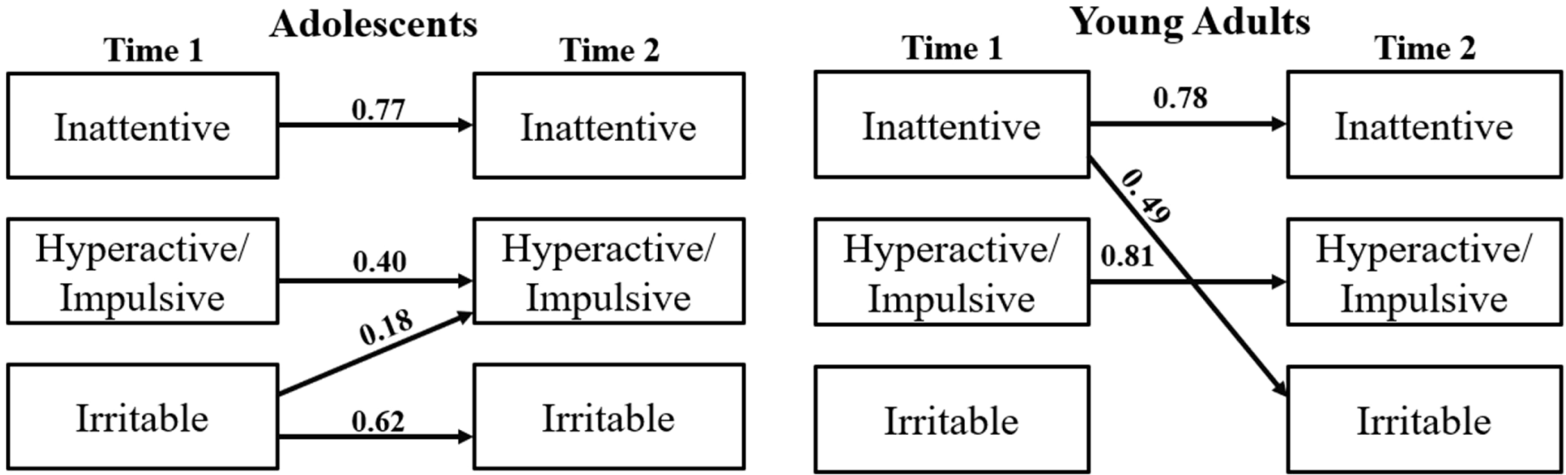
Multivariate regression analysis. adolescents (AIC = -429.032, RMSEA = 0.113), young adults (AIC = -343.244, RMSEA = 0.107).

**Figure 2** shows an overview of the final model and its performance metrics for both adolescents and young adults. We can see that in adolescents, irritability at T1 is associated with hyperactivity/impulsivity at T2 (β = 0.18, p-value < 0.001), and in young adults, inattentiveness is associated with irritability in later years (β = 0.49, p-value < 0.05).

Further, we extended the analysis to study potential sex differences. We observed that in adolescent females, irritability predicted higher hyperactive-impulsive scores in later years (β = 0.26, p-value < 0.001), whereas in male adolescents no significant correlation emerged.

In young adult females, inattentiveness was a predictor variable for irritability (β = 0.49, p-value < 0.05). During subgroup analysis for young adults, we observed that the multivariate regression model for males exhibited instability. This instability may be attributed to differences in the distribution of covariates among males compared to females, potentially affecting model convergence and robustness.

**Figure 3:**
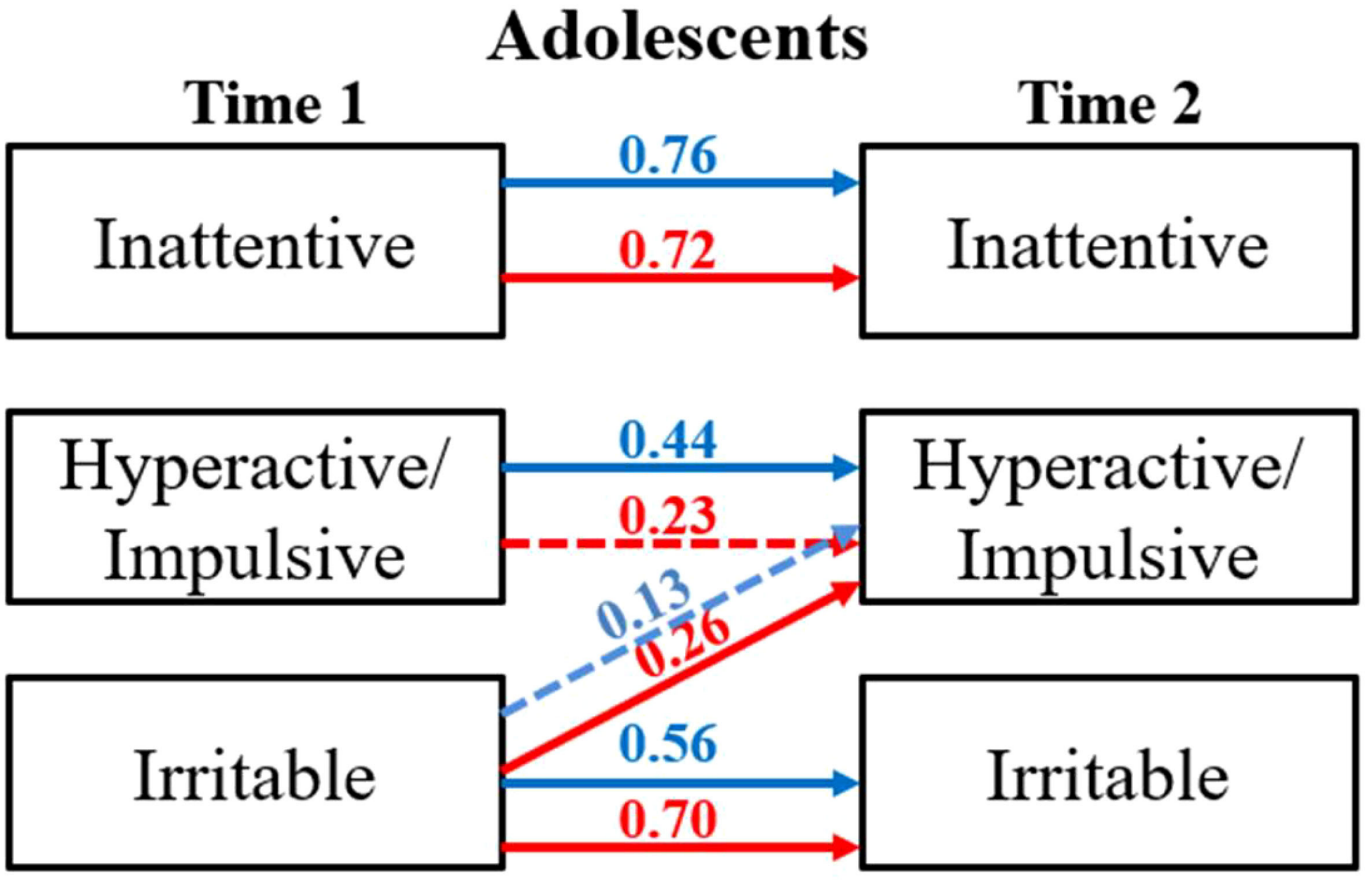
Multivariate regression analysis: group analysis. red female, blue male. Significant paths (p-value < 0.05) are solid red/blue lines and non-significant paths (p-value > 0.05) are depicted using dashed red/blue lines.

The results, shown in **Figure 3**, confirm our previous findings (34) regarding the stability of the link between irritability and hyperactive/impulsive symptoms in adolescent females and underscore the importance of considering sex differences in the study of irritability and its impact on ADHD-related symptoms.

### 3.2 Machine learning analysis

The ML analysis was exclusively conducted on adolescent females, as this subgroup was where irritability predicted hyperactivity/impulsivity within our dataset. We utilized behavioral items to construct ADHD symptoms and the irritability subscale at T1, and investigated the predictability of hyperactive/impulsive symptoms at T2. Before inputting these features into the model, hierarchical clustering was applied to all variables, comprising nine items for hyperactivity/impulsivity, nine items for inattentiveness, and five items for irritability at T1. Using features selected by the clustering algorithm, we trained a non-parametric model employing RF analysis to evaluate the significance of predictability in hyperactive/impulsive scores at T2.

#### 3.2.1 Multicollinearity and hierarchical clustering

**Figure 4:**
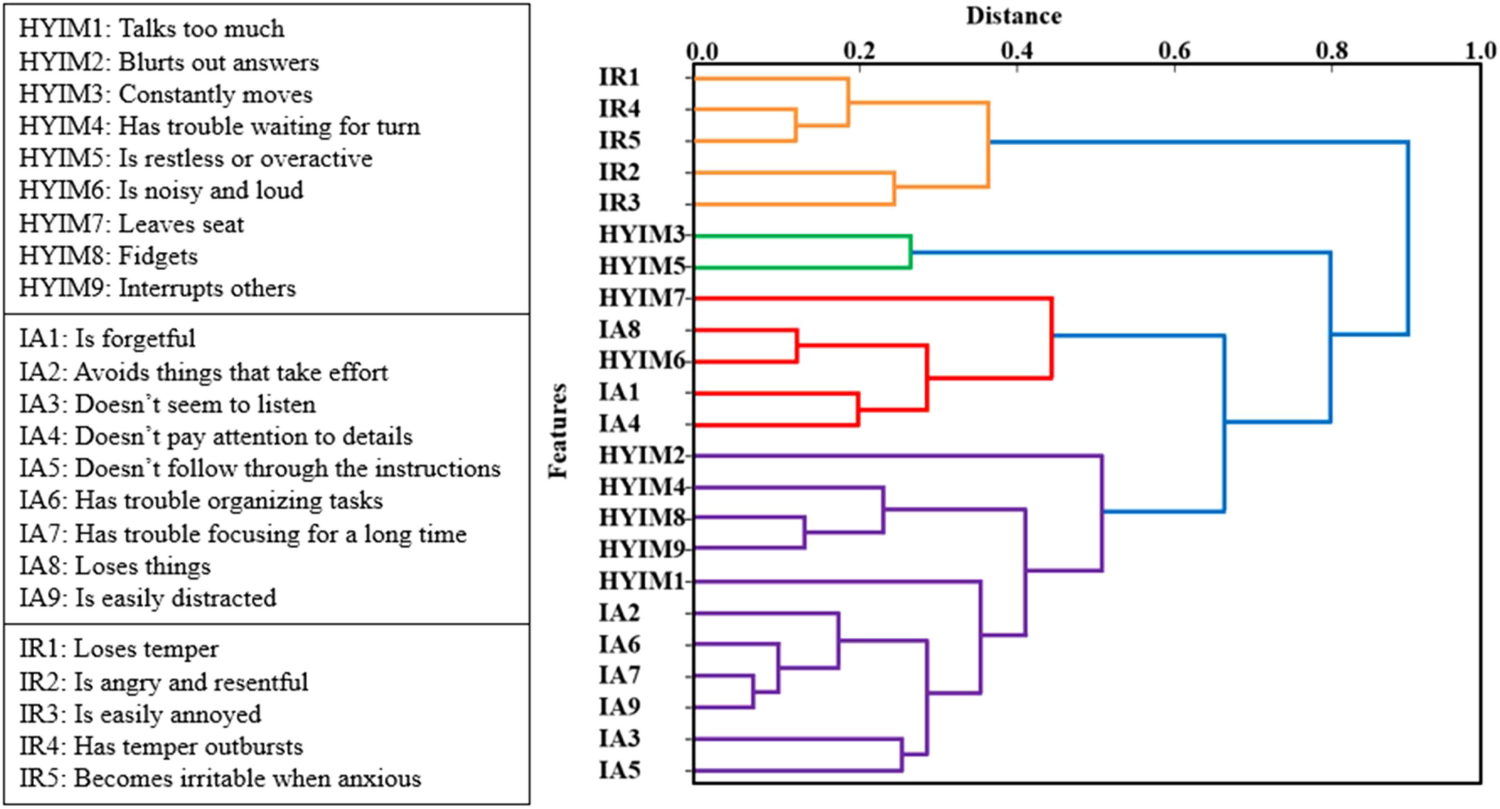
The dendrogram of features includes ADHD constructs and irritability subscale scores at the behavior level in adolescent females. The x-axis represents the similarity between clusters while the y-axis represents the features. Each U-shaped structure in the tree denotes a cluster, with two branches fusing at a specific level of similarity. The length of the U-shape indicates the distance between them. With the threshold set to 0, each variable is treated as an individual cluster.

**Figure 4** shows the dendrogram resulting from applying our clustering algorithm based on Ward’s linkage method to the dataset, illustrating how features are organized into clusters at various levels of similarity.

In this dendrogram, the y-axis represents the data points or features, while the x-axis represents the dissimilarity (distance). As we ascend the tree (from left to right), branches merge, leading to an increase in the distance. Each U-shaped structure in the tree represents a cluster, with two branches fusing at a specific level of similarity. The length of the U-shape indicates the distance between them as considered as a threshold for clustering.

With the threshold set to 0, each variable is treated as an individual cluster. Increasing the threshold in 0.1 increments from 0 to 1, we had more variables emerge in each cluster resulting in a smaller number of clusters and less correlation among them.

**Figure 5:**
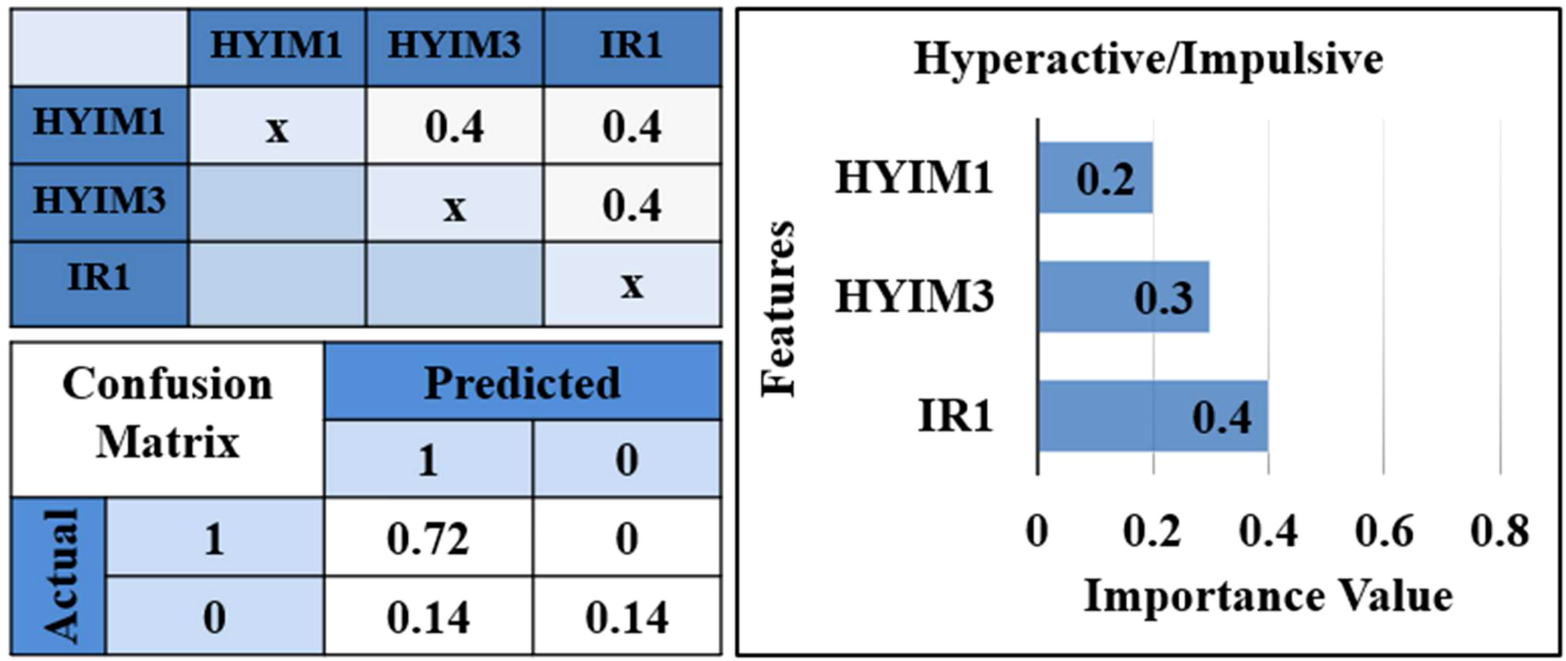
Correlation matrix, confusion matrix, and features’ importance. Correlation matrix for selected features from each cluster while the clustering threshold is set to 0.7. Confusion matrix for the test set for predicted values. *IR1: “loses temper”, HYIM1: “talks too much”, HYIM3: “constantly moves”.

To eliminate the impact of highly correlated variables and their influence on the results, the threshold was increased to 0.7. At this threshold, the clusters included the following items from the rating scale: [hyim1, hyim2, hyim4, hyim6, hyim7, hyim8, hyim9, ia1, ia2, ia3, ia4, ia5, ia6, ia7, ia8, ia9], [hyim3, hyim5], and [ir1, ir2, ir3, ir4, ir5]. As shown in **Figure 5**, the correlation among the clusters chosen at threshold 0.7 was negligible. This clustering technique effectively addressed multicollinearity, allowing for more robust modeling and accurate estimation of features’ importance. From threshold 0 to 1, the restructuring of clusters led to improved accuracy scores. At threshold 0.7 we had the highest performance vs thresholds lower or higher.

In each model, we replaced the selected features with alternative variables within the same cluster. In each iteration, the performance showed slight fluctuations and the importance of the features remained relatively consistent. Ultimately, features yielding higher performance from each cluster were chosen to integrate into the final model.

#### 3.2.2 Model configuration and hyperparameters

The RF classifier was configured using GridSearchCV and these hyperparameters were eventually chosen to balance model complexity and performance, aiming to prevent overfitting: number of estimators: 10, maximum depth: 3, minimum samples of each split: 2, minimum samples of each leaf: 1, maximum number of features: ‘sqrt’, and bootstrap: True.

#### 3.2.3 Model performance metrics

The performance of the RF classifier on the test set is summarized by the following metrics:

- Accuracy (86%): The model correctly predicted 86% of the instances.
- Precision (100%): All positive predictions made by the model were correct, indicating no false positives.
- Recall (50%): The model identified 50% of the actual positive instances.
- F1 Score (66.7%): This is the harmonic mean of precision and recall, providing a single metric that balances the trade-off between the two.

#### 3.2.4 Confusion matrix

The confusion matrix provides a detailed breakdown of the model’s classification performance:

- True Negatives (TN): 72% of the negative instances were correctly classified.
- False Positives (FP): 0% of the negative instances were incorrectly classified as positive.
- False Negatives (FN): 14% of the positive instances were incorrectly classified as negative.
- True Positives (TP): 14% of the positive instances were correctly classified.

This reveals that the model is highly precise in identifying positive instances (no false positives).

#### 3.2.5 Feature importances

**Figure 5** shows irritability item 1, “loses temper” selected from the cluster including all irritability items, has the highest importance 0.4 in predicting hyperactive/impulsive symptom significance at T2. The third hyperactive/impulsive item, “constantly moves” is the second significant feature with a value of 0.3, selected from the cluster including the third and fifth hyperactive/impulsive symptoms. The first hyperactive/impulsive item, “talks too much” selected from the cluster including all inattentive items and hyperactive/impulsive items except the third and fifth is the third highest feature with a value of 0.2.

#### 3.2.6 Prediction model for impulsive symptom

**Figure 6:**
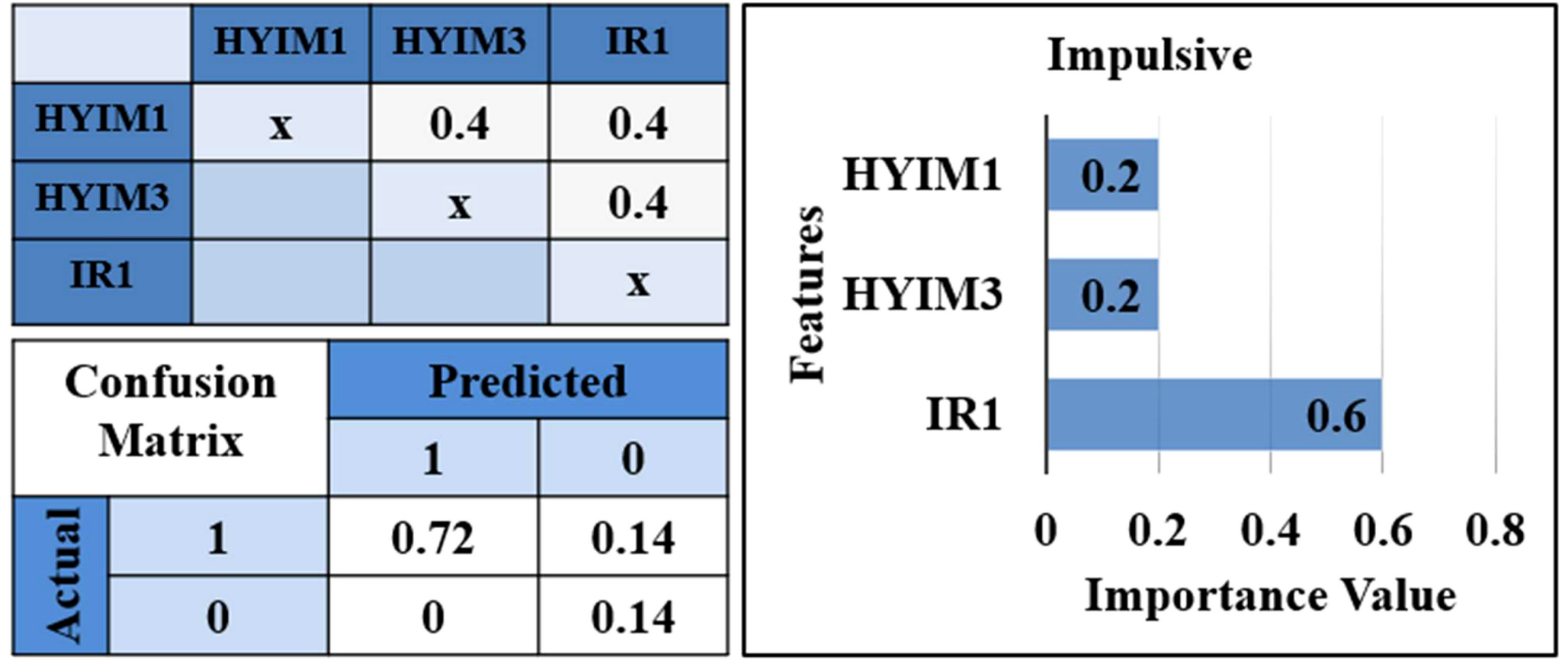
Correlation matrix, confusion matrix, and features**’** importance for impulsive prediction model. *IR1: “loses temper”, HYIM1: “talks too much”, HYIM3: “constantly moves”.

Further, we separated the scores for impulsivity and hyperactivity symptoms and used features selected based on hierarchical clustering to train two different classifiers. As **Figure 6** shows, the results showed irritability had a high ranking in the prediction of impulsive symptoms (accuracy: 86%, precision: 100%, recall: 50%, f1: 66.7%) and the performance of the prediction model for the hyperactive model was significantly lower.

## 4 Discussion

Irritability is not a diagnostic symptom of ADHD, but individuals diagnosed with ADHD often display intense, negative emotional reactions to minor setbacks and challenges (66–68). This study extended our previous work which investigated the relation between irritability and ADHD symptoms in adolescents using the Conners’ Parent Rating Scale-3 (34). We augmented our dataset by including Conners’ self-rating scale for adolescents and expanded the study for young adults using the CAARS self and observer rating scales. In the longitudinal study, we explored sex-based differences and the role of irritability in predicting ADHD symptoms. Using multivariate regression analysis, we identified a significant link between irritability at T1 and hyperactive-impulsive symptoms at T2 within adolescent females.

We introduced a novel approach; a machine-learning model coupled with a hierarchical clustering technique. This approach helped us illuminate the relationships between items within each subscale. We performed a fine-grained analysis by including all compounding behaviors of each symptom and applied Ward’s linkage method and RF to cluster the variables, remove collinearities, and predict symptom significance at a later time. The performance of our model was significantly high, and it showed irritability items have a relatively higher importance in predicting hyperactive/impulsive symptoms for adolescent females compared to adolescent males and young adults. This approach was particularly crucial, given the constraints of our relatively small sample size.

### 4.1 Irritability-impulsivity link and sex differences

Analyzing the results of two distinct models for impulsivity and hyperactivity, we observed that in adolescent females, irritability has a higher rank in identifying impulsive symptoms than hyperactive symptoms. This irritability-impulsivity link may be due to underlying challenges with emotional regulation, related to brain structure and function and perhaps with dopaminergic regulation of activity during this critical sensitive period of brain development (69, 70). Indeed, irritable moods and frustration may contribute to heightened impulsive reactions (71). Also, the significant association between irritability and impulsiveness among females may arise from the challenges, uniquely experienced by females with ADHD, where the presence of irritability could increase difficulty in regulating their emotions, resulting in more impulsive behaviors (72). It may also be that if they are experiencing irritability related to the omission of expected rewards, they may seek out rewards in the future, including ones that are more immediately available, thus acting impulsively, to ameliorate a deficit in a reward state as those with higher irritability may have an increased sensitivity to both omission and receipt of rewards (73). A mediation analysis in future research could clarify this correlation. Fluctuations in hormonal functioning during these periods may also be relevant (74). Future studies should investigate potential links between daily and monthly hormonal fluctuations and irritable, hyperactive/impulsive, and inattentive symptoms in adolescents with ADHD. This area of inquiry is beyond the scope of our project, as our data collection focused on overall pubertal development rather than specific hormonal patterns. Future analyses could also explore whether the higher ratings on emotional constructs, such as irritability, may reflect bias toward, females than males in relation to societal expectations (75). In addition, future work should integrate behavioral and functional neuroimaging data to investigate emotional and cognitive regulation. This approach may produce less bias than rating scale measures, providing objective data to complement studies that rely on potentially biased rating scales. The association between irritability and hyperactivity/impulsivity symptoms in females, but not males, suggests higher stability in irritability ratings for females. Stability may be a conserved phenomenon in females due to the way dysregulated mood progresses in females versus males, or due to differences in the etiology of attention in females versus males. It is also likely that there are genetic origins of brain regional sex differentiation that are related to externalizing vs internalizing symptoms (76).

### 4.2 Irritability-inattentiveness association in young adults

In adulthood, we found inattention predicted a subsequent higher likelihood of irritability. Inattention could be associated with lower peer acceptance and victimization and can predict irritability (77). Individuals who struggle with maintaining attention might find it harder to connect with their peers and may be more vulnerable to being targeted or mistreated by others. These negative social experiences, such as rejection or bullying, can then lead to heightened irritability as a reaction to the stress and frustration caused by these interpersonal challenges. Also, this connection might be attributed to the known association of inattention to functional impairments. Tasks requiring prolonged focus can be emotionally draining, especially in groups where there are already higher levels of inattention, such as in our study, contributing to higher irritability (78). The implication is that individuals who struggle with maintaining attention for extended periods may experience increased feelings of irritability as a result of the added emotional strain brought about by these tasks.

### 4.3 Machine learning approach

We extended our previous work (34) and validated our results using multivariate regression. However, it is important to acknowledge that linear regression, being a parametric model, assumes linearity in relationships, the absence of multicollinearity, and a normal distribution of data. Here we use a non-parametric approach that unlike linear regression does not make any assumption about data distribution showing that irritability items have higher importance in predicting hyperactive-impulsive symptoms in adolescent females.

Machine learning approaches can be top-down hypothesis-driven bottom or data-driven. The latter can be unstructured and exploratory, and prone to false positives, overfitting, and fitting to noise. Larger samples help avoid this issue. However, hypothesis-driven approaches – such as our present study - are powered by expert domain knowledge, in the form of published clinical findings, thus narrowing the problem search domain and feature space.

Machine learning approaches benefit from larger sample sizes and future studies should attempt to include larger samples. Our dataset initially comprised of 54 instances, which was further reduced to 33 due to missing data from participants at T2. We conducted a statistical test on all variables at T1 between two groups: those with second-time data and those without. Our observation revealed no significant difference between the groups concerning the variables selected as input to the model (IR1, HYIM1, and HYIM3), indicating that these variables share the same distribution.

Our modest sample size was able to harness the strengths of an extremely well-characterized community-recruited longitudinal sample of adolescents with and without ADHD, with deep behavioral phenotyping and multimodal imaging. This sample has been carefully recruited to include participants with the Combined Presentation Type of ADHD and without comorbid disorders such as autism, learning disabilities, or severe mental conditions at Time 1 (e.g., psychosis, depression, or substance use disorders) to limit confounding variables. We also excluded youth during T1 with comorbid disorders because a primary goal of the overall project from which these data were taken was to assess the emergence of later psychiatric diagnoses, such as depression, anxiety, posttraumatic stress disorder (PTSD), and substance use disorders in relation to earlier impulsivity symptoms and ADHD diagnoses. However, the exclusion of volunteers with comorbid disorders such as depression may have limited the generalizability to youth who have both diagnosable depression and ADHD. Our ADHD group did have higher overall depressive symptoms than the NT group (34), though not to the degree that the symptoms met criteria for a diagnosis of depression.

In our approach, we offered a solution in the form of feature engineering, aimed to simplify the model and enhance performance. In a model with a large number of highly collinear features, we utilized a hierarchical clustering approach, represented visually through a dendrogram, to identify clusters of variables and similarities among them. By aggregating highly correlated features into the same cluster and selecting one representative feature from each cluster, we ensured that the feature importance of the new model remained unaffected by collinearity.

Having collinear features and clustering them results in less intuition about the interpretability of the model. However, hierarchical clustering had the advantage of providing a detailed view of relations between features at different levels of granularity to enhance model performance (79). Remarkably, hierarchical clustering showed that all irritability items were found in one cluster, validating its high internal consistency as a construct. In addition, all inattentive and hyperactive/impulsive items, except hyim3 (i.e., constantly move) and hyim5 (i.e., restless and overactive), form another cluster suggesting that most adolescent females exhibit either a combined presentation of ADHD symptoms or no symptoms at all. This observation aligns with the corresponding statistics for this group, where 12 out of 33 participants show a combined presentation of symptoms, 18 show no symptoms, and three displayed a few symptoms but were not diagnosed with ADHD. These findings underscore the applicability of this approach in gaining deeper insight into data distribution and remind us of the transdiagnostic nature of irritability.

The chosen hyperparameters reflect a trade-off between complexity and performance. The max_depth of 3 and n_estimators of 10 suggest a relatively simple model, which helps in preventing overfitting given the small dataset size. The high precision but low recall indicates that the model is conservative in making positive predictions. This results in fewer false positives but at the cost of a higher number of false negatives. The balanced F1 score reflects the need to improve recall without significantly sacrificing precision. The confusion matrix underscores the model’s strength in correctly identifying negative instances and the distribution of feature importances suggests that all features contribute meaningfully to the model’s predictions, with Feature 3 (hyim3) being the most influential.

In summary, our proposed framework can be an important foundation within the data analysis process, particularly when preparing data for an ML model. This approach deepened our understanding of the data, discovered the collinearity between features, and made informed decisions about feature selection for our prediction model. Moving forward, our approach can be expanded to analyze larger datasets, employing irritability as either a predictor or outcome variable across various dimensions of ADHD and functional outcomes and further enhancing precision diagnostics and treatment decisions.

## Data Availability

The datasets used in this study are available from the National Data Archive and from Dr. Schweitzer.
